# Gut microbiota genome features associated with brain injury in extremely premature infants

**DOI:** 10.1080/19490976.2024.2410479

**Published:** 2024-10-07

**Authors:** David Seki, Rasmus Kirkegaard, Jay Osvatic, Bela Hausmann, Joana Séneca, Petra Pjevac, Angelika Berger, Lindsay J Hall, Lukas Wisgrill, David Berry

**Affiliations:** a Centre for Microbiology and Environmental Systems Science, Department of Microbiology and Ecosystem Science, Division of Microbial Ecology, University of Vienna, Vienna, Austria; b Joint Microbiome Facility of the Medical University of Vienna and the University of Vienna, Vienna, Austria; c Intestinal Microbiome, School of Life Sciences, ZIEL - Institute for Food & Health, Technical University of Munich, Freising, Germany; d Department of Laboratory Medicine, Medical University of Vienna, Vienna, Austria; e Department of Pediatrics and Adolescent Medicine, Division of Neonatology, Pediatric Intensive Care and Neuropediatrics, Comprehensive Center for Pediatrics. Medical University of Vienna, Vienna, Austria; f Food Microbiomes and Health, Quadram Institute Bioscience, Norwich, UK; g Norwich Medical School, University of East Anglia, Norwich, UK; h Microbes, Infections, and Microbiomes, Institute of Microbiology and Infection, University of Birmingham, Birmingham, UK

**Keywords:** Perinatal white matter injury, *Enterobacteriaceae*, gut-microbiota-brain axis, extremely premature infants, siderophores, inflammation, nitrate, nanopore metagenomics

## Abstract

Severe brain damage is common among premature infants, and the gut microbiota has been implicated in its pathology. Although the order of colonizing bacteria is well described, the mechanisms underlying aberrant assembly of the gut microbiota remain elusive. Here, we employed long-read nanopore sequencing to assess abundances of microbial species and their functional genomic potential in stool samples from a cohort of 30 extremely premature infants. We identify several key microbial traits significantly associated with severe brain damage, such as the genomic potential for nitrate respiration and iron scavenging. Members of the *Enterobacteriaceae* were prevalent across the cohort and displayed a versatile metabolic potential, including pathogenic and nonpathogenic traits. Predominance of *Enterobacter hormaechei* and *Klebsiella pneumoniae* were associated with an overall loss of genomic functional redundancy as well as poor neurophysiological outcome. These findings reveal microbial traits that may be involved in exacerbating brain injury in extremely premature infants and provide suitable targets for therapeutic interventions.

## Introduction

Extremely premature infants frequently suffer from severe brain damage, which is thought to involve pathological development of the gut-microbiota-immune-brain axis.^1^ In healthy individuals, the commensal gut microbiota participates in the establishment of homeostatic conditions that facilitate absorption of nutrients and promotion of immunological tolerance, which are both critical for postnatal neurophysiological expansion and refinement.^2^ However, injuries of the central nervous system can induce systemic inflammation and thereby compromise initial assembly of gastrointestinal (GIT) microbiota, favoring subsequent blooms of opportunistic pathobionts that can contribute to poor neurodevelopmental outcomes and life-long morbidity.^3^

Although the postnatal assembly of the gut microbiota in extremely premature infants has been extensively described by taxonomic profiling,^4–7^ we lack a detailed understanding of microbial traits underlying primary ecological succession during this critical developmental window. Generally, initial GIT community assembly and primary succession is characterized by colonization of facultative anaerobes transitioning to an increased presence of strictly anaerobic bacteria in premature^3^ as well as in term-born infants.^6^ In this transition, it is thought that *Bifidobacterium* spp. are key beneficial symbionts, as they play a role in host metabolism,^8^ immunological homeostasis,^9^ and protection against pathogen invasion.^10^ However, abundances of *Bifidobacterium* spp. are often low in extremely premature infants, which instead have high loads of *Enterobacteriaceae*. ^11^ Blooms of *Enterobacteriaceae* have been associated with sepsis and necrotizing enterocolitis, and members of this family are often considered to be opportunistic pathogens or pathobionts.^12^

Severe brain damage constitutes a major perturbation of the gut-brain axis, with brain and intestinal development being closely intertwined. Vagal afferents sense aberrant alterations of GIT microbiota and project information of distress to various forebrain regions to induce an appropriate parasympathetic activation in response,^13^ which includes inflammatory priming of immune cells and their migration to the intestine and meninges.^14^ Moderate inflammatory hits typically involve anti-inflammatory feedback loops to reestablish homeostasis once the inflammatory source is cleared. However, recurring and chronic inflammation of either intestinal or cerebral origin can block such auto-regulatory feedback-loops, thus potentiating the release of pro-inflammatory signals and respective receptor activities, leading to increased GIT permeability and pro-inflammatory activity.^15^ It is postulated that in response to this inflammatory environment microbiota adjust their physiology to survive, and that these adjustments might be linked to further aggravations of brain injury.^16^

Here, we report on a metagenomic analysis of extremely premature infant gut microbiota, linking species-resolved taxonomy and respective metabolic potentials to the pathology of perinatal white matter injury, as diagnosed via cranial magnetic resonance imaging at term-equivalent age. We find that an overall loss in genetic functional redundancy as well as enrichment in iron scavenging and nitrate reduction pathways are key genomic traits associated with proliferation of putative pathobionts that may be involved in aggravating severe brain damage.

## Material and methods

### Experimental model, clinical definitions, stool sample and clinical data collection

Extremely premature infants were enrolled between September 2017 and June 2019 at the General Hospital of Vienna/Medical University of Vienna as part of the PreMiBraIn study,^3^ which was approved by the ethics committee of the Medical University of Vienna (ethics number 1348/2017). Inclusion criteria were birth before the 28th week of gestation with less than 1,000 g birth weight. Infants with congenital malformations, chromosomal aberrations, maternally transmitted infectious diseases (e.g., HIV, hepatitis A/B/C), and inborn errors of metabolism were excluded. Clinical parameters of each patient were prospectively monitored by the clinical staff during hospitalization and recorded within the hospitals’ electronic database, ICIP (Philips Healthcare Systems). All monitored clinical parameters are listed in Table 2 and Supplementary File 1. For parameters with continuous monitoring or multiple measurements per day (e.g. FiO_2_, IL-6 screenings), we calculated daily averages per patient.

Routine cranial magnetic resonance imaging (cMRI) screenings at term-equivalent age were assessed for brain damage via established scores,^3,17^ as well as expert radiologist knowledge. Diagnosed severe brain injuries included periventricular hemorrhagic infarctions, intraventricular hemorrhages, cerebellar hemorrhages, and periventricular leukomalacia. Many of these pathologies were also accompanied by a reduction of brain volume and an enlargement of the subarachnoid spaces. Starting from the first day of life every infant received the probiotic preparation Infloran (*B. bifidum* and *L. acidophilus*). The enteral feeding regimen includes the infant’s own mother’s milk or pasteurized human donor milk. Stool samples were collected from all infants over the course of hospitalization, and of these, 63 samples were picked for metagenomic sequencing. This resulted in a set of samples from 24 neonates without severe brain damage (*n* = 50 samples) and 6 neonates with severe brain damage (*n* = 13 samples), with a minimum of 6, and a maximum of 57 days post-delivery. Each stool was sampled from diapers via collection tubes during patient care routines and immediately stored at − 80°C until further analysis.

### Isolation of DNA and metagenomic sequencing

DNA was extracted using the Power Soil Pro Kit (Qiagen) following the manufacturer’s protocol for stool samples. Additionally, we used total nucleic acid (TNA) extracts with sufficiently high quality from previous extracts,^3^ which were weighed in (20–30 mg) for phenol-chloroform extraction of DNA,^18^ with the inclusion of one bead-beating step. DNA was eluted in 40 μl nuclease free water and stored at −20°C until further analysis. DNA concentration was measured using the HSdsDNA Assay Kit (Thermo Fisher) on a Qubit 4 Fluorometer. For Oxford Nanopore long-read sequencing, DNA was prepared using the rapid barcoding sequencing kit (SQK-RBK110-96, Oxford Nanopore Technologies) following the manufacturers protocol. DNA was sequenced on a Promethion P24 (Oxford Nanopore Technologies) on a R9.4.1 flowcell (FLO-PRO002, Oxford Nanopore Technologies) using Minknow (v. 20.10.3, Oxford Nanopore Technologies). Reads were basecalled using Guppy (v. 5.0.12) using super accuracy mode. For Illumina short-read sequencing, barcoded libraries were prepared using the NEBNext Ultra II FS Library Prep Kit for Illumina (NEB) and sequenced on a NovaSeq 6000 platform (v1.5 chemistry, 300 cycles, 2 × 150bp)

### Read-based processing of sequencing data

Short-read metagenomic data (Illumina) were processed using HUMAnN 3^19^ with default setting. Briefly, HUMAnN 3 first estimates community composition with MetaphlAn 4,^20^ second it maps reads to a community pangenome with bowtie2^21^ and third it aligns unmapped reads to a protein database using DIAMOND.^22^ This results in both taxonomic as well as functional profiles of the metagenome reads. For the functional profiles, reads were mapped to MetaCyc pathway definitions to group sets of pathways. Counts of taxonomic profiles as estimated in relative abundances via MetaphlAn 4 were multiplied by total cell counts as determined via qPCR previously,^3^ and species counts were corrected for the number of rRNA operons.^23^

### De novo assembly and processing of metagenome-assembled genomes (MAGs)

The nanopore reads were assembled using flye (v. 2.8.3-b1695),^24^ followed by polishing of the assembly once via racon using short-reads with the aligner minimap2 (v. 2.17)^25^ and racon (v. 1.4.3),^26^ followed by two rounds of polishing with medaka (v. 1.4.1, github.com/nanoporetech/medaka) using long-reads. The Illumina reads were trimmed using cutadapt (v. 3.1).^27^ Reads were mapped to the assemblies using minimap2 (v. 2.17),^25^ read mappings were converted using samtools (v. 1.11)^28^ for binning with metabat2 (v. 2.15).^29^ The quality of the binned MAGs was assessed using QUAST (v. 5.0.2),^30^ CheckM (v. 1.1.1),^31^ they were taxonomically classified using GTDBtk (v. 2.1.0),^32^ and functionally annotated using Prokka.^33^ The MAG dataset was dereplicated with standard settings via dRep,^34^ and the remaining representative MAGs were classified as either pathobionts or commensals by a wide scope pathogenicity classifier.^35^ Bbmap^36^ was used to calculate the percentage of illumina reads mapping to dereplicated MAG. Furthermore, ABRicate – db NCBI (https://github.com/tseemann/abricat.) was used to screen for antimicrobial resistance genes,^37^ and FeGenie^38^ was used to screen for iron-related genes in dereplicated MAGs. Lastly, we used METABOLIC with METABOLIC-C.pl for the classification of the metabolic capabilities of dereplicated genomes, and their respective contribution to metabolic processes.^39^

### Statistical analysis and data visualization

Statistical analysis (Student’s T-test, ANOVA, repeated measures ANOVA, Wilcoxon Test, and Fisher’s exact test) were performed in R version 4.0 and the R package rstatix version 0.7.0. Hierarchical clustering was performed with ComplexHeatmap^40^ on log-transformed abundance counts, and DESeq2^41^ was used for differential abundance testing of microbial species. All p-values were adjusted using Bonferroni’s method. Data was visualized via R version 4.0 and R package ggplot2 version 3.3.3.^42^

## Results

### Species-level composition of the gut microbiota in extremely premature infants

The pathology of perinatal white matter injury is multifarious, and its relation to gut microbiota remains elusive. In a previous study, we found that aberrant development of the gut-immune-brain axis post-delivery is predictive for the extent of subsequent brain damage.^3^ Here, we metagenomically characterize stool samples (*n* = 63 samples) collected during the initial 60 days post-delivery from a subset of extremely premature infants (gestational age <28 weeks, and birth weight <1 kg) from the same cohort. We analyzed samples from 24 (*n* = 50 samples) infants without severe brain damage, and 6 (*n* = 13 samples) which suffered from severe brain damage, to gain a deeper understanding of the microbiota at species level resolution. Detailed information regarding the timing of sampling per patient can be found in Figure S1).

**Figure 1.**
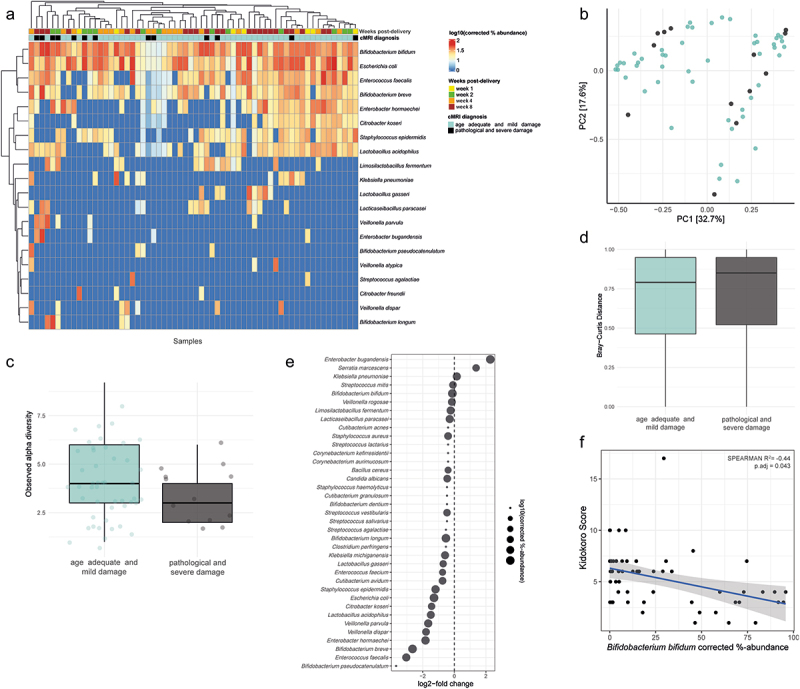
Species-level composition of the gut microbiota in extremely premature infants.

We used short metagenomic reads to assign taxonomy via Metaphlan4,^20^ and corrected for compositionality via 16S rRNA gene-targeted qPCR. For this, we averaged microbial abundances per patient to exclude repeated measures, and we excluded reads that mapped to *Candida albicans*, which was the only non-bacterial member of the gut microbiome detected but was only found sporadically and at < 1% relative abundance. *Escherichia coli* (*E. coli*) and *Bifidobacterium bifidum* (*B. bifidum*) were the most abundant bacterial species across the patient cohort, partially completely dominating individual gut communities. Hierarchical clustering of samples did not indicate grouping due to neurophysiological outcome (Figure 1(a) and Table 1). Also, multivariate analysis of variance (PERMANOVA) as well as principal component analysis (PCA) of absolute species abundance revealed no significant relationship between cMRI diagnosis or days of life post-delivery on microbiome composition (PERMANOVA, p.adj = 0.49; Figure 1(b) and 1(c)). Likewise, no significant differences in observed alpha diversity were detected between infants with and without severe brain damage (t test, p.adj = 0.113; Figure 1d). We next evaluated whether there were differentially abundant species between infants with and without severe brain damage using DESeq2.^41^ Among prevalent species, *Enterobacter bugandensis* (*E. bugandensis*) had the largest average enrichment in infants with severe brain damage and *Enterococcus faecalis* (*E. faecalis*) the largest average depletion, although these trends were not significant after correction for multiple testing (Figure 1(e)).

Turquoise color for age-adequate cranial magnetic resonance imaging (cMRI) results or mild brain injury. Black color for severe brain injury. a) Hierarchical clustering of log-transformed absolute abundance-corrected counts of twenty most abundant bacterial species, as determined by short-read classification of illumina reads via MetaPhlan4. b) Principal component analysis (PCA) of absolute species abundance count. c) Boxplots summarizing Bray–Curtis distances of pairwise comparisons within infants with severe brain damage and infants without. d) Microbial richness measured by the count of unique bacterial species per patient group. e) Differential abundance testing via DESeq2 for the assessment of differentially abundant microbial species between infants with and without severe brain damage. f) Spearman correlation between the cMRI assessment measure “Kidokoro score” and corrected %-abundances of *Bifidobacterium bifidum*.

**Table 1.** Abundance distribution of microbiota.

Species	Average: age adequate and mild damage	Average: pathological and severe damage	Prevalence: Age adequate and mild damage	Prevalence: pathological and severe damage	Minimum: age_adequate and mild damage	Minimum: pathological and severe damage	Maximum: age adequate and mild damage	Maximum: pathological and severe damage
*Bifidobacterium bifidum*	30	29.85	87.76	84.62	1.2	5.01	94.51	95.68
*Bifidobacterium breve*	7.36	3.05	26.53	15.38	1.66	3.33	74.13	36.09
*Bifidobacterium longum*	0.06	6.1	2.04	7.69	2.82	79.33	2.82	79.33
*Bifidobacterium pseudocatenulatum*	1.56	0	6.12	0	15.64		36.24	
*Candida albicans*	0.75	0.12	8.16	7.69	1.47	1.52	27.14	1.52
*Citrobacter freundii*	2.03	0	2.04	0	99.58		99.58	
*Citrobacter koseri*	1.31	0.01	6.12	0	5.25		30.04	
*Enterobacter bugandensis*	0	4.2	0	15.38		22		32.59
*Enterobacter hormaechei*	6.57	10.11	18.37	23.08	1.06	10.83	94.42	98.16
*Enterococcus faecalis*	6.93	0.82	42.86	15.38	1.46	2.81	96.85	6.47
*Escherichia coli*	35.19	28.6	65.31	61.54	1.13	1.27	99.82	77.86
*Klebsiella michiganensis*	0.29	0.15	2.04	7.69	14.39	2	14.39	2
*Klebsiella pneumoniae*	1.07	0.38	6.12	7.69	2.03	4.88	36.69	4.88
*Lactobacillus acidophilus*	2.6	0.25	16.33	7.69	1.13	2.6	93.6	2.6
*Lactobacillus gasseri*	0.66	0.21	6.12	7.69	1.09	2.73	16.68	2.73
*Limosilactobacillus fermentum*	0.62	0.69	12.24	15.38	1.25	1.72	20.39	7.27
*Staphylococcus epidermidis*	0.3	7.04	4.08	7.69	3.8	90.9	4.08	90.9
*Veillonella atypica*	0.58	0	2.04	0	27.94		27.94	
*Veillonella dispar*	0.42	2.83	6.12	7.69	5.08	36.81	8.51	36.81
*Veillonella parvula*	0.33	4.81	4.08	7.69	3.08	62.04	12.86	62.04

Routine clinical measurements of systemic inflammation, hepatic dysfunction, and impaired respiration were associated with cMRI diagnosis in the patient cohort (Table 2 and supplementary file 1). We therefore evaluated whether any microbial species were associated with clinical measurements. This revealed significant correlations between overall food-balance-intake and *Lacticaseibacillus paracasei* (*L. paracasei*; SPEARMAN = 0.43, p.adj = 0.047), and between body-mass index and *L. paracasei* (SPEARMAN = 0.434 p.adj = 0.035) and *Streptococcus salivarius* (*S. salivarius*; SPEARMAN = 0.46, p.adj = 0.018). Lastly, Kidokoro scores, a measure for severity of brain damage via conventional MRI screenings at term-equivalent age, were negatively correlated with *B. bifidum* abundances (SPEARMAN = −0.44, p.adj = 0.043; Figure 1(f)), indicating that *B. bifidum* abundance is associated with healthy neurophysiological outcome.

**Table 2.** Clinical parameters of subject cohort.

Description	group1	group2	n1	n2	mean_group1	mean_group2	statistic	df	p	p.adj	p.adj.signif
Bilirubin direct [mg/dl]	age_adequate_and_mild_damage	pathological_and_severe_damage	375	169	0.66186667	0.60443787	1.26990519	431.215054	205	736	ns
Bilirubin total [mg/dl]	age_adequate_and_mild_damage	pathological_and_severe_damage	548	244	2.91458029	2.91053279	0.02025283	420.382235	984	984	ns
Bilirubin conjugated [mg/dl]	age_adequate_and_mild_damage	pathological_and_severe_damage	376	169	0.0606117	0.0087574	3.77101076	470.988579	0.000183	0.001464	**
Bilirubin unconjugated [mg/dl]	age_adequate_and_mild_damage	pathological_and_severe_damage	376	169	2.32175532	2.49319527	−0.75845641	292.499552	449	898	ns
GOT (ASAT) [U/L]	age_adequate_and_mild_damage	pathological_and_severe_damage	522	242	30.8582375	29.9793388	0.59553101	558.820056	552	984	ns
GPT (ALAT) [U/L]	age_adequate_and_mild_damage	pathological_and_severe_damage	519	232	29.0260116	27.3771552	2.1430901	706.993515	0.0324	0.1944	ns
LDH [U/L]	age_adequate_and_mild_damage	pathological_and_severe_damage	310	133	336.317742	390.815789	−2.95006756	176.777707	0.00361	0.02527	*
gamma-GT [U/L]	age_adequate_and_mild_damage	pathological_and_severe_damage	467	215	91.4143469	83.544186	1.2311041	436.761745	219	736	ns
Headsize	age_adequate_and_mild_damage	pathological_and_severe_damage	896	193	23.59252	23.51813	0.50187579	291.89048	616	616	ns
Weight	age_adequate_and_mild_damage	pathological_and_severe_damage	1859	729	1.7913128	1.5651721	1.19970522	1081.18639	231	231	ns
Length	age_adequate_and_mild_damage	pathological_and_severe_damage	1860	729	39.5642	38.32247	3.7467277	1206.91029	0.000188	0.000188	***
BMI	age_adequate_and_mild_damage	pathological_and_severe_damage	1859	729	1.066312	1.0032	−0.78554006	855.340802	432	432	ns
IL-6	age_adequate_and_mild_damage	pathological_and_severe_damage	455	200	161.9357	124.8302	0.6006912	652.321884	548	548	ns
CRP	age_adequate_and_mild_damage	pathological_and_severe_damage	368	185	1.864021	1.378459	1.73164623	540.498266	0.0839	0.0839	ns
FiO2	age_adequate_and_mild_damage	pathological_and_severe_damage	896	444	31.08141	32.61166	−3.23476537	1019.85413	0.00126	0.00126	**
SaO2	age_adequate_and_mild_damage	pathological_and_severe_damage	1701	673	92.33238	92.0841	1.87101725	1152.43478	0.0616	0.0616	ns
ART: BE [mmol/L]	age_adequate_and_mild_damage	pathological_and_severe_damage	193	43	−3.814498	−2.506047	−2.55068897	85.5593015	0.0125	0.05	*
ART: SBC [mmol/L]	age_adequate_and_mild_damage	pathological_and_severe_damage	193	43	20.55591	21.281008	−1.75656561	82.161479	0.0827	0.2481	ns
ART: pCO2 [mmHg]	age_adequate_and_mild_damage	pathological_and_severe_damage	193	43	49.684397	55.192303	−3.17169824	66.6194948	0.00229	0.01145	*
ART: pH	age_adequate_and_mild_damage	pathological_and_severe_damage	193	43	7.273594	7.261567	1.16673327	65.059129	248	496	ns
ART: pO2 [mmHg]	age_adequate_and_mild_damage	pathological_and_severe_damage	193	43	56.528068	55.345244	0.73184565	71.73574	467	496	ns
CAP: BE [mmol/L]	age_adequate_and_mild_damage	pathological_and_severe_damage	145	50	0.577046	1.4947333	−1.20122142	91.2861893	233	372	ns
CAP: SBC [mmol/L]	age_adequate_and_mild_damage	pathological_and_severe_damage	143	49	23.7965967	24.7062245	−1.25874515	74.0917836	212	372	ns
CAP: pCO2 [mmHg]	age_adequate_and_mild_damage	pathological_and_severe_damage	145	50	53.5487241	56.2344667	−1.53109701	104.542872	129	0.27642857	ns
CAP: pH	age_adequate_and_mild_damage	pathological_and_severe_damage	145	50	7.3130706	7.308477	0.42903325	82.5124804	669	0.71678571	ns
CAP: pO2 [mmHg]	age_adequate_and_mild_damage	pathological_and_severe_damage	145	50	40.0968276	36497	2.24992935	165.813255	0.0258	0.118125	ns
VEN: BE [mmol/L]	age_adequate_and_mild_damage	pathological_and_severe_damage	248	65	−0.1780578	0.3862637	−0.88466172	111.742865	378	0.51545455	ns
VEN: SBC [mmol/L]	age_adequate_and_mild_damage	pathological_and_severe_damage	248	64	22.8890726	23.0449405	−0.28851937	111.200416	773	773	ns
VEN: pCO2 [mmHg]	age_adequate_and_mild_damage	pathological_and_severe_damage	248	65	57.5007056	60.5184615	−1.63631499	91.5616731	105	0.2625	ns
VEN: pH	age_adequate_and_mild_damage	pathological_and_severe_damage	248	65	7.2768921	7.2711498	0.48917843	87.3457702	626	0.71678571	ns
VEN: pO2 [mmHg]	age_adequate_and_mild_damage	pathological_and_severe_damage	247	64	37.4475709	34.8510789	2.16541076	205.043941	0.0315	0.118125	ns
Calcium ionized (BGA) [mmol/L]	age_adequate_and_mild_damage	pathological_and_severe_damage	481	116	1.343421	1.326238	1.55517368	162.835498	122	327	ns
Chlorid (BGA) [mmol/L]	age_adequate_and_mild_damage	pathological_and_severe_damage	481	116	109.061239	106.238701	3.46354889	176.686813	0.000669	0.003345	**
Glucose (BGA) [mg/dl]	age_adequate_and_mild_damage	pathological_and_severe_damage	482	116	116.781508	132.727607	−3.70689121	158.768164	0.000289	0.001734	**
Potassium (BGA) [mmol/L]	age_adequate_and_mild_damage	pathological_and_severe_damage	481	116	4.422151	4.330875	1.61050412	221.282191	109	327	ns
Lactate (BGA) [mmol/L]	age_adequate_and_mild_damage	pathological_and_severe_damage	481	115	1.562652	1.777361	−2.57701459	152.473766	0.0109	0.0436	*
Sodium (BGA) [mmol/L]	age_adequate_and_mild_damage	pathological_and_severe_damage	481	116	138.620444	137.616626	1.57545204	176.619204	117	327	ns
Albumin [g/L]	age_adequate_and_mild_damage	pathological_and_severe_damage	207	41	25.0756039	25.9853659	−1.41299174	60.3945728	163	502	ns
Alkaline Phosphatase [U/L]	age_adequate_and_mild_damage	pathological_and_severe_damage	147	36	373.70068	337.305556	1.15803877	63.6414017	251	502	ns
Anorganic Phosphate [mmol/L]	age_adequate_and_mild_damage	pathological_and_severe_damage	130	24	1.9072308	1.8541667	0.639142	30.3038898	528	704	ns
Urea-N [mg/dl]	age_adequate_and_mild_damage	pathological_and_severe_damage	126	25	22.1150794	21622	0.18494994	32.6663073	854	915	ns
Creatinin [mg/dl]	age_adequate_and_mild_damage	pathological_and_severe_damage	187	40	0.5472193	0.605375	−1.77825223	51.7254686	0.0812	502	ns
Magnesium [mmol/L]	age_adequate_and_mild_damage	pathological_and_severe_damage	123	22	0.9510569	0.9268182	0.76166515	31.3760873	452	704	ns
Protein [g/L]	age_adequate_and_mild_damage	pathological_and_severe_damage	140	28	41.3071429	42.4642857	−1.26319295	46.0627948	213	502	ns
Triglyceride [mg/dl]	age_adequate_and_mild_damage	pathological_and_severe_damage	162	35	140.975309	144.142857	−0.10791231	43.1484112	915	915	ns
Total volume	age_adequate_and_mild_damage	pathological_and_severe_damage	970	384	140.45	143.595234	−1.43239685	642.45282	153	0.19125	ns
Ratio parenteral	age_adequate_and_mild_damage	pathological_and_severe_damage	970	384	63.182474	59.955729	2.15745706	712.896566	0.0313	0.0586875	ns
Ratio enteral	age_adequate_and_mild_damage	pathological_and_severe_damage	970	384	36.817526	40.044271	−2.15745706	712.896566	0.0313	0.0586875	ns
Total energy	age_adequate_and_mild_damage	pathological_and_severe_damage	970	384	103.224856	103.963958	−0.45089141	677.789455	652	0.69857143	ns
Total carbs	age_adequate_and_mild_damage	pathological_and_severe_damage	970	384	8.402711	8.293021	0.87493391	670.900544	382	0.44076923	ns
Total protein	age_adequate_and_mild_damage	pathological_and_severe_damage	970	384	4.097546	4.102786	−0.09117919	673.908601	927	927	ns
Total fat	age_adequate_and_mild_damage	pathological_and_severe_damage	970	384	4.167093	4.331989	−1.737861	682.972866	0.0827	0.11277273	ns
Parenteral energy	age_adequate_and_mild_damage	pathological_and_severe_damage	970	384	52.686103	48.534818	3.33136744	718.080445	0.000908	0.00681	**
Parenteral carbs	age_adequate_and_mild_damage	pathological_and_severe_damage	970	384	5.006363	4.568921	4.08347694	696.309253	0.0000495	0.0007425	***
Parenteral protein	age_adequate_and_mild_damage	pathological_and_severe_damage	970	384	2.542268	2.386458	2.74147289	694.646899	0.00627	0.03135	*
Parental fat	age_adequate_and_mild_damage	pathological_and_severe_damage	970	384	1.364797	1.262808	1.80679955	739.919152	0.0712	0.1068	ns
Enteral energy	age_adequate_and_mild_damage	pathological_and_severe_damage	970	384	50.538928	55.429349	−2.17462624	692.085454	0.03	0.0586875	ns
Enteral carbs	age_adequate_and_mild_damage	pathological_and_severe_damage	970	384	3.396356	3.723769	−2.21366478	692.426488	0.0272	0.0586875	ns
Enteral protein	age_adequate_and_mild_damage	pathological_and_severe_damage	970	384	1.555273	1.716484	−1.95857183	686.267792	0.0506	0.08433333	ns
Enteral fat	age_adequate_and_mild_damage	pathological_and_severe_damage	970	384	2.802338	3.069096	−2.19299937	693.675586	0.0286	0.0586875	ns

### Altered functional potential and reduced functional redundancy are associated with brain damage

**Figure 2.**
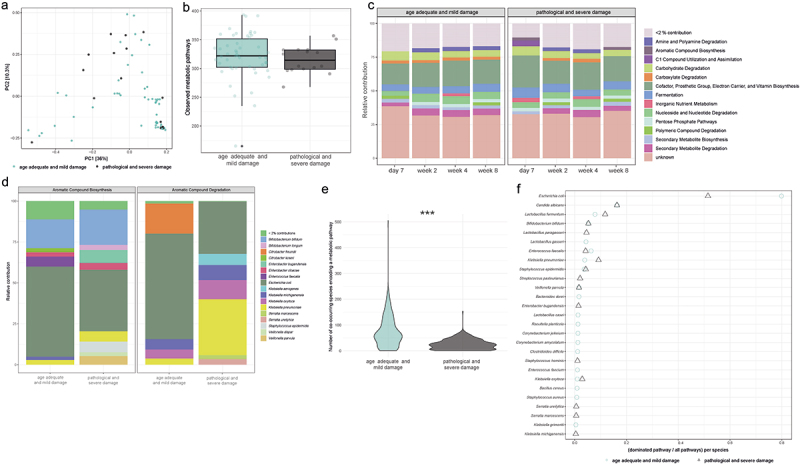
Functional potential of gut microbiota in extremely premature infants.

To identify the functional potential of the infant gut microbiota during the first month post-delivery, we employed read-based annotation of genes via HUMAnN 3.^19^ Thereby, metabolic pathway abundances for the whole community as well as species-specific pathways were evaluated. We found no effects of cMRI diagnosis on compositionality of community-level pathway profiles (PERMANOVA, p.adj >0.05; Figure 2(a)) or on the number of metabolic pathways detected (t.test, p.adj >0.05, Figure 2(b)). Next, we aggregated metabolic pathways into broader nutrient metabolism categories, finding that at eight weeks post-delivery in microbiota of infants with severe brain damage significantly more reads were attributed to aromatic compound degradation (t test, p.adj = 0.009; Figure 2(c)), with most of these reads mapping to *Klebsiella pneumoniae* (*K. pneumoniae*; Figure 2(d)). We also observed a lowered contributions of pathways for sucrose degradation III (sucrose invertase) as well as for starch degradation III by *E. coli*, but these observations were not statistically significant after correction for multiple testing (t.test, p.adj = 0.25 and 0.44, respectively; Figure S2).

Turquoise color for age-adequate cranial magnetic resonance imaging (cMRI) results or mild brain injury. Black color for severe brain injury. a) Principal component analysis (PCA) of community-level metabolic pathway abundance, as determined by short-read classification of illumina reads via HUMAnN3. b) Functional richness measured by counting unique metabolic pathways per patient group. c) Relative abundance of community-level metabolic pathways throughout hospitalization, aggregated into broader MetaCyc-categories. d) Relative read contributions of microbial species to metabolic pathways in infants with and without severe brain damage. e) Violin-plots displaying the number of co-occurring species that encode the same metabolic pathway in infants with severe brain damage. f) Count of dominated metabolic pathways (>51% read contributions) per respective species. Asterisks represent adjusted *p* values: *p.adj <0.05, **p.adj <0.01, ***p.adj <0.001.

Functional redundancy is thought to facilitate stability of the adult gut microbiota,^43^ but little is known concerning the redundancy of metabolic pathways in the human infant gut microbiota. Although the number of detected metabolic pathways did not significantly differ with disease diagnosis (Figure 2(b)), metabolic pathways tended to be encoded by fewer co-occurring species in infants with severe brain damage (t-test, p.adj <0.001; Figure 2(e)). To further explore this loss of functional redundancy, we evaluated pathway dominance by a single species, defined as a single species accounting for ≥51% of reads for a given metabolic pathway. With this definition, we observed an overall increase in the number of pathways dominated by *Lactobacillus fermentum (L. fermentum)* and *K. pneumoniae* and a reduction in the number of pathways dominated by *E. coli* and *E. faecalis* in infants with severe brain damage (Figure 2(f)).

In summary, we find indications for associations between severe brain damage, aromatic compound degradation via *K. pneumoniae*, as well as participation in nutrient utilization pathways by several bacterial species. Furthermore, functional redundancy is severely reduced in infants with brain damage, with some *Enterobacteriaceae* such as *K. pneumoniae* dominating a broad range of functional pathways accompanied by the loss of metabolic pathway dominance by *E. faecalis* and other *Enterobacteriaceae* including *E. coli*.

### Identification of genomic features characteristic for pathobionts and commensals

**Figure 3.**
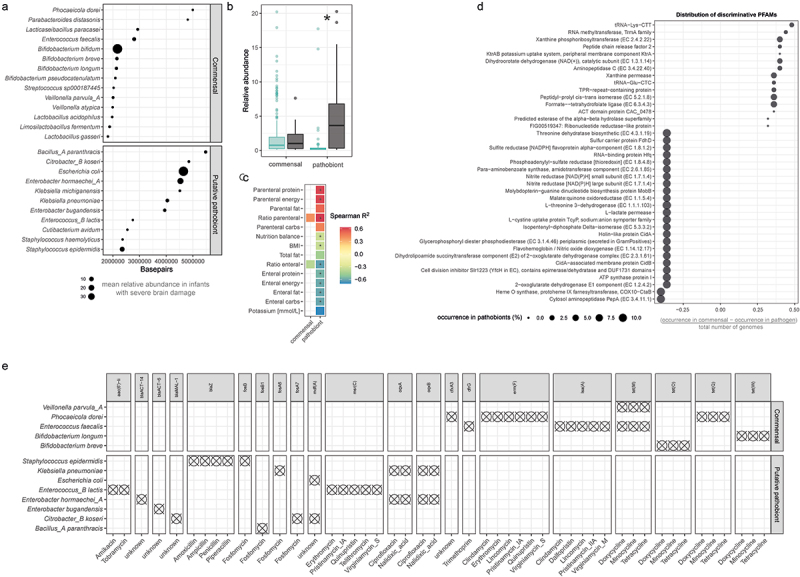
Genetic traits of pathobionts and commensals.

Putative pathobionts are defined as species that are regular members of the gut microbiota that can drive pathologies following intestinal perturbation. To identify traits that define commensal or pathobiont microorganisms in the gut microbiota of extremely premature infants, we assembled genomes from metagenomic reads, yielding 25 unique, highly complete (>90%), and high-quality (<5% contamination) bacterial MAGs after dereplication, allowing for robust linkage between genomic potential and species identity. This set of MAGs covered 65,16% (±15,23%) of all reads in infants with severe brain damage, and 61,79% (±16,14%) in infants without. Next, we employed the machine-learning based model “wide scope pathogenicity classifier” (WSPC).^34^ This model classifies genomes as either commensal or putative pathobionts based on their entire protein-coding content, integrating hypothetical as well as annotated protein families (PFAMs) (Figure 3a). According to this classification, putative pathobionts on average had higher relative abundances in infants with severe brain damage (t.test, p.adj = 0.037; Figure 3b). We next correlated relative abundances of MAGs grouped as putative pathobionts or commensals against all available routine clinical examination data. This revealed significant correlations between pathobiont abundance and the ratio of overall parenteral nutrition (SPEARMAN = 0.63, p.adj = 0.005), parenteral protein (SPEARMAN = 0.61, p.adj = 0.01), parenteral energy (SPEARMAN = 0.61, p.adj = 0.01), overall food-balance-intake (SPEARMAN = −0.33, p.adj = 0.02), enteral protein (SPEARMAN = −0.57, p.adj = 0.03), enteral energy (SPEARMAN = −0.6 p.adj = 0.01), enteral fat (SPEARMAN = −0.61 p.adj = 0.01), enteral carbohydrates (SPEARMAN = −0.61, p.adj = 0.01), and the ratio of overall enteral nutrition (SPEARMAN = −0.63 p.adj = 0.005) (Figure 3c).

a) Dot plots displaying genome size of dereplicated metagenome assembled genomes (MAGs). MAGs were sorted by wide scope pathogenicity classification, and size of dots indicates mean relative abundance of given MAG in infants with severe brain damage. b) Boxplots displaying average relative abundance of commensals and putative pathobionts in infants with (with) and without (turquoise) severe brain damage. c) Spearman correlation between average abundances of commensals or putative pathobionts and routine clinical examination data. d) Dot plot displaying protein families (PFAMs) that are significantly associated with either commensal or pathobiont genomes, as identified by Fisher-Exact test. Size of dots indicates relative occurrence of respective PFAM in pathobiont genomes. e) Dot plot visualizing the presence of antibiotic resistance genes in dereplicated MAGs, as screened via ABRicate. Asterisks represent adjusted *p* values: *p.adj <0.05.

We next sought to identify which protein-coding genes underlie differentiation of putative pathobiont and commensal MAGs. Although the proportion of PFAMs with unknown function was higher in commensals (FISHER.EXACT.test, p-value <0.001), most protein families of both groups were at least partially annotated (87% and 77% of all PFAMs respectively; Figure S3A) and found at diverging probabilities in respective MAGs (Figure S3B and S3C). Several PFAMs were significantly associated with either commensals or pathobionts (Figure 3d). Notably, lactate and nitrite utilization genes (L-lactate permease and large and small subunit for nitrite reductase) were detected as putative pathobiont traits, and the xanthine utilization genes as a commensal trait.

Antibiotics are commonly administered in intensive care units. Notably, we find that premature infants with brain damage have been administered cefuroxime six times more frequently than premature infants without (Table 3). MAGs carried several antibiotic resistance genes (ARGs), but no resistance for cefuroxime. Overall, we detected ARGs in 13 out of 25 genomes (Figure 3e). Interestingly, commensal and pathobiont MAGs encoded distinct ARG profiles. Several commensal microbes, including *Veillonella parvula (V. parvula)*, *Phocaeicola dorei (P. dorei)*, *E. faecalis*, *Bifidobacterium longum (B. longum)*, and *B. breve*, encoded ARGs against doxocycline, minocycline, and tetracycline, and *E. faecalis* additionally had ARGs against clindamycin, dalfopristin, lincomycin, pristinamycin, and virginiamcyin. Conversely, pathobiont MAGs encode ARGs against amikacin, tobramycin, amoxicillin, ampicillin, penicillin, piperacillin, fosfomycin, erythromycin, pristinamycin, quinupristin, telithromycin, virginiamycin, ciproflaxin, and nalidixic acid. Most notably, *K. pneumoniae* and *E. hormaechei* (*E. hormaechei*) both encoded ARGs against ciprofloxacin and nalidixic acid, and *K. pneumoniae* additionally possessed fosfomycin resistance.

**Table 3.** Frequency of antibiotic administration in subject cohort.

Antibiotic	number of administrations in infants without severe brain damage	number of administrations in infants with severe brain damage	frequency of administration in infants without severe brain damage	frequency of administration in infants with brain damage
Ampicillin	76	28	2	1.866666667
Azithromycin	5	0	0.131578947	0
Cefuroxim	5	34	0.131578947	2.266666667
Ciprofloxacin	2	0	0.052631579	0
Gentamicin	153	59	4.026315789	3.933333333
Meropenem	45	12	1.184210526	0.8
Piperacillin/Tazobactam	99	30	2.605263158	2
Vancomycin	16	2	0.421052632	0.133333333

In a recent study, we found associations between several metabolites and severe brain damage in infants in this cohort via untargeted metabolomics.^44^ We therefore screened MAGs for the presence of genes involved in the processing of these manually selected neuroactive metabolites (bile acids, butanoate, cholic acid, ethanol/methanol, fatty acids, galactitol, indoles, kynurenine, mevalonate, nicotine, porphyrins, propane, sialic acids, steroids, and tryptophan) to evaluate their respective distribution among commensals and putative pathobionts. Notably, we find that the potential for utilization of ethanolamines as well as porphyrins and propane are well represented among *K. pneumoniae*, *Klebsiella michiganensis (K. michiganensis)*, *E. coli*, and *Citrobacter koseri (C. koseri)*, but not among many commensal bacteria (Figure S3D).

We found that putative pathobionts were elevated in infants with severe brain injury, and that pathobiont MAGs were enriched in several protein families, including L-lactate permease and both subunits for nitrite reductase, which may be important to survival in the GIT under chronic inflammatory conditions.^16^ The pathobionts *K. pneumoniae* and *E. hormaechei* also stand out due to their antibiotic resistance profile, including ARGs conferring resistance to ciproflaxin and fosfomycin, both of which are commonly administered to premature infants.^45^

### Elevated potential for nitrate reduction and iron scavenging by putative pathobionts in infants with severe brain damage

**Figure 4.**
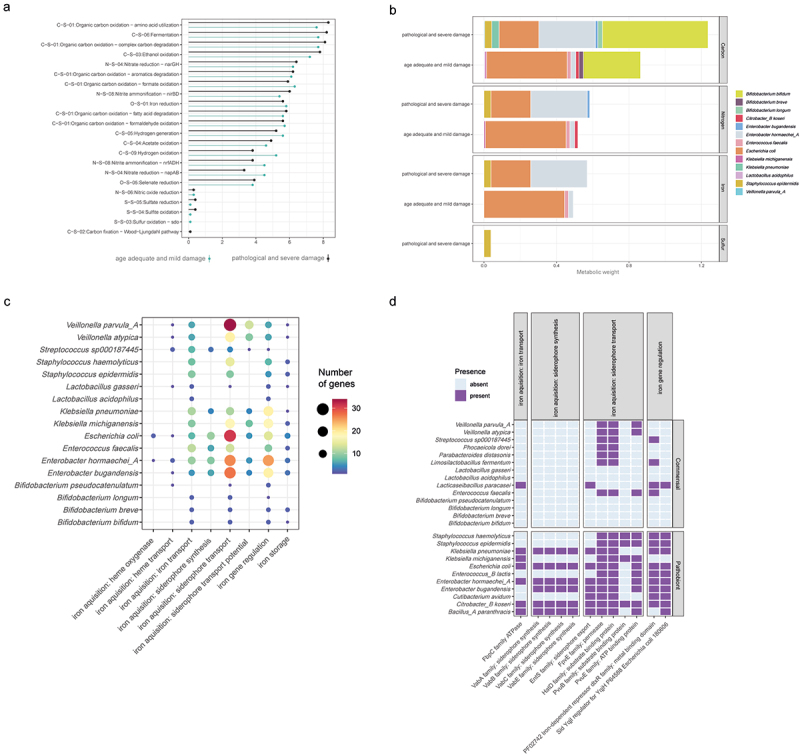
Microbial metabolism in premature infants with severe brain damage.

We further investigated differences in microbial metabolic potentials in infants with and without severe brain damage and calculated species-level contributions to biochemical transformations using METABOLIC.^38^ Briefly, species-level contributions reflect the functional capacity and abundance of the given species within the network of other co-occurring microorganisms. Several pathobionts possessed metabolic potentials for 4-aminobutyrate aminotransferase and related aminotransferases, or phenol transformation, but no such functions were found in commensal bacteria. Genes for urea degradation, oxygen reduction, nitrate and nitrite reduction, perchlorate reduction, chlorite reduction, formate oxidation, and formaldehyde oxidation were well represented in putative pathobiont MAGs but not in many commensals (Figure S4A). The abundance of biochemical transformation classes did not significantly differ between infants with and without severe brain damage (Figure 4a). On a species level however, we found increased contributions to overall carbon cycles by *Staphylococcus epidermidis (S. epidermidis)*, *K. pneumoniae*, *E. hormaechei*, *E. bugandensis*, *V. parvula*, *B. bifidum*, and *B. longum*. Notably, *B. longum* had an elevated potential for oxidation of ethanol in infants with severe brain damage. Furthermore, *S. epidermidis*, *E. hormaechei*, and *E. bugandensis* displayed elevated contributions to overall nitrogen cycles. By contrast, we observed a reduction of *E. coli* contribution to overall carbon cycles and a complete loss of contributions from *B. breve*, *K. michiganensis*, *Lactobacillus acidophilus (L. acidophilus)*, *C. koseri*, and *E. faecalis* in infants with severe brain damage. In premature infants with pathological brain development, we observe an elevated sulfur metabolism potential due to the genetic potential for sulfite oxidation by *S. epidermidis*. Most strikingly, in infants with severe brain damage, we observe an increase in the potential for *E. hormaechei* to reduce Fe^3+^, whereas in premature infants with healthy brain development that metabolic function is mainly encoded by *E. coli* and *E. faecalis* (Figure 4b and Figure S4B).

Metabolic potentials in metagenome assembled genomes (MAGs) were investigated on community and species-level via METABOLIC. a) Dotplot visualizing community-level abundances of biochemical transformation classes in infants with and without severe brain damage. b) Bar charts visualizing summed metabolic weights and contributions of respective species toward given biochemical cycle categories. c) Count of iron-related genes in dereplicated MAGs d) Heatmap displaying presence or absence of iron-related genes, which are significantly associated with either commensal or pathobiont genomes, as identified by Fisher–Exact test.

We next employed FeGenie^37^ to further analyze iron processing genes in MAGs and found that genes involved in iron acquisition are broadly distributed across both commensal and pathobiont genomes. *Enterobacteriaceae* possesses multiple genes for the regulation and accession of iron via siderophores, though there were inter-species differences in gene copy numbers. *E. faecalis* has comparatively fewer iron-related genes and lacks genes for siderophore transport potential, whereas *K. michiganensis*, *Veillonella atypica (V. atypica)*, and *V. parvula* lack genes for siderophore synthesis (Figure 4c). However, multiple iron-related genes are enriched in putative pathobionts (FISHER.EXACT.test, respective p-values <0.001; Figure 4d). Genes for synthesis of alternative siderophores were predominantly found in putative pathobionts, and some siderophore transport genes were found in *Bifidobacterium* species (Figure S5).

In conclusion, we found that pathobionts have terminal reductases for oxic and anoxic respiration, as well as an extensive siderophore gene repertoire. *E. hormaechei* can partially displace the closely related *E. coli* under inflammatory conditions related to brain injury. Its potential to secure bioavailable iron appears to be of crucial importance. The commensals *B. longum* and *B. bifidum* remain present under these inflammatory conditions and contribute to the functional potential of the gut microbiota, particularly in the degradation of complex carbohydrates.

## Discussion

Here, we address GIT microbiome composition and metabolic traits that are associated with severe brain damage in extremely premature infants (born before 28 weeks of gestation with <1 kg). Compared to previous studies, we find consistent levels of *Proteobacteria*, *Firmicutes*, and *Actinobacteria* being reflective of the major microbial phyla in extremely premature infants^3^ as well as low-birth-weight premature infants.^46^ However, we achieved better taxonomic resolution via long-read Nanopore sequencing on microbial species-level, especially for the *Enterobacteriaceae*, a diverse family of putative human pathogens. Additionally, by using and cross validating several metagenomic approaches, we find that pathobionts are cumulatively more abundant in premature infants with neuropathological outcomes. They possess a versatile genomic potential that facilitates survival during chronic inflammation, including multiple respiratory pathways, many antibiotic resistance genes, and several means of iron acquisition.

Potential pathobionts in the GIT of extremely premature infants mainly include *Enterobacteriaceae* such as *E. hormaechei* and *K. pneumoniae*. In the genomes of both, we found resistance to ciprofloxacin, an antibiotic that was, however, rarely administered. The three most frequently administered antibiotics were piperacillin, gentamicin, and ampicillin (Table 3), but resistance to these was only found in *Staphylococcus*. We also found tetracycline resistance (TetR) genes in commensal *B. longum* and B. *breve*, which are reportedly carriers of TetR, but in which these genes are not mobile, therefore deemed safe for probiotic use.^47^ It is widely accepted that bifidobacteria are beneficial to health,^9^ but it remains neglected whether they perform relevant functions during pathological processes, during which they persist as well (Table 1). We observed that metabolic weights (as defined in the work by Zhou et al.^39^ of *B. longum* toward oxidation of ethanol are increased in infants with severe brain damage (Figure S4B), indicative for an interaction with *Enterobacteriaceae*, which via mixed-acid fermentation are the main potential producers of ethanol in the GIT.^48^ Furthermore, we speculate that ethanol removal via *B. longum* may be essential for the preservation of GIT integrity, given the toxicity of ethanol for developing colonocytes.^49^

Chronic inflammation in premature infants with severe brain damage is driven by proliferation of inflammatory T-cells whilst persisting intestinal occupation via pathobionts, at an expense in production of neuroprotectants,^3^ resulting epithelial damage and excess generation of reactive oxygen species (ROS) in the gastrointestinal tract.^50^ Ferrous iron is transformed to less accessible ferric states in the presence of ROS, thus organisms evolved numerous mechanisms to retain this vital resource in anaerobic environments.^51^ The metabolic weights of pathogens in extremely premature infants are shifted toward the acquisition of iron during aberrant development of the gut-immune-brain axis (Figure 4b). These pathobionts are known to secrete siderophores to chelate and monopolize iron, upon which host immune cells secrete siderophore-binding proteins such as lactoferrin, and thereby further exacerbate pro-inflammatory cytokine secretion.^52^ Notably, lactoferrin is broadly administered in combination with probiotic supplementation in premature infants, to favor host-absorption of iron. However, several iron-acquisition genes were significantly associated with putative pathobionts, including alternative siderophores potentially resistant to iron restriction via lactoferrin (Figure 4d). *E. hormaechei* and *K. pneumoniae* are recently emerging pathogens that pose an epidemiological threat,^53^ especially due to the clustered antimicrobial resistance they carry (Figure 3e), and therefore alternatives to antibiotics aiming to reduce the fitness of pathogenic *Enterobacteriaceae* during infection and inflammation are urgently needed.

The “tug-of-war” for survival over iron supplies between host and pathobionts during inflammation offers promising targets for such alternatives, as for instance the development of siderophore-conjugated antibiotics acting as “trojan-horses”,^54^ or the introduction of deactivating siderophore-binding competitors to Fe^3+^, such as Gallium (Ga^3+^).^55^ Manganese (Mn^3+^) well could be considered, given that commensal *Lactobacillus* spp. completely relies on manganese instead of iron,^56^ but hardly establish in the GIT, despite daily probiotic supplementation of *L. acidophilus*. However, supplementation of manganese might prove detrimental as many opportunistic pathogens can employ manganese as an alternative to iron for functioning enzymes.^57^ Other commensals of the premature infant gut do however depend on iron, and we lack therapeutic options that facilitate recovery of commensal traits and balanced homeostatic conditions. Polyphenols could serve for this purpose, since they combine iron-chelating as well as ROS-suppressing qualities,^58^ and polyphenol-chelated iron would in theory remain degradable by a broad microbial spectrum, given that traits for phenol degradation are more widely distributed than alternative siderophore secretion, while simultaneously alleviating burdens of inflammation.

Besides accumulation of ROS, further intestinal consequences of inflammation involve re-orientation of damaged colonocytes toward an inflammatory-skewed metabolism that is characterized by elevated anaerobic glycolysis, involving less consumption of oxygen and higher release of lactate, oxygen, nitrate, and nitric oxide into the lumen, despite the availability of intracellular oxygen and glucose.^16^ As opposed to fermentation, nitrate reduction yields more energy and is therefore preferentially used when available.^59^ Being a key trait of pathobionts (Figure 3d), *Enterobacteriaceae* encode both nitrate and nitrite reduction genes (Figure S4A) and exploit this niche for energy during inflammation induced via severe brain damage (Figure 4b). This could provide a competitive advantage over other microbiota lacking the potential for nitrate reduction and lead to overgrowth of pathobionts, which could furthermore prohibit reestablishment of non-inflammatory metabolic states of colonocytes,^60^ thus underlying further aggravations of severe brain damage.^15^ Therefore, nitrite or nitrate reductases serve as promising target enzymes for the development of alternative antimicrobials with the potential to reduce inflammation and alleviate aggravations of brain damage in premature infants. A common feature shared by N-compound utilizing terminal reductases is the incorporation of an essential molybdenum cofactor into their active sites, and therefore a suppression in nitrate respiration upon molybdenum deficiency.^61^ Tungsten could be considered for therapeutic interventions since it competes for binding in the nitrate reductase complex but results in inactivation of the enzyme once it replaced the molybdenum.^61^ Tungsten oxide nanoparticles were recently used to reduce dysbiotic blooms of *Enterobacteriaceae* during acute colitis in mice.^62^ Due to its specificity, tungsten is only operational on enzymes that allow for nitrate reduction during inflammation but exerts only small influence during homeostatic conditions,^63^ and tungsten supplementation could therefore be considered as an additional prophylactic measure.

We conclude that a loss of functional redundancy emerges during the pathology of severe brain damage in extremely premature infants. Our data suggest that survival of putative pathobionts such as *E. hormaechei* and *K. pneumoniae* is facilitated by its potential for utilization of nitrite and nitrate, as well as scavenging of iron during gastrointestinal inflammation, thereby perhaps out-competing other *Enterobacteriaceae* and further exacerbating perinatal white-matter injury. These findings suggest novel metabolic targets for alternative therapeutic interventions and may hold potentials to restore homeostatic primary succession of microbiota and protect premature infants from severe brain damage.

## Supplementary Material

Figure_S1.tiff

Figure_S2.tiff

Figure_S5.tiff

Suppl_File_1.docx

Figure_S4.tiff

Figure_S3.tiff

STORMS_Seki_2024.docx

## Resource availability

### Lead contact

Further information and requests for resources and reagents should be directed to and will be fulfilled by the lead contacts David Berry (david.berry@univie.ac.at) and David Seki (david.seki@univie.ac.at).

### Materials availability

Deposition of materials under BioProject ID: PRJNA1112760

## Teaser

Nanopore sequencing reveals microbial features that are linked to the pathology of perinatal white-matter injury in extremely premature infants.
