# Supplementary figures and images for: Gut microbiota genome features associated with brain injury in extremely premature infants
Source: Gut Microbes. 2024 Oct 7;16(1):2410479. doi: 10.1080/19490976.2024.2410479 (PMC11459832; doi:10.1080/19490976.2024.2410479)

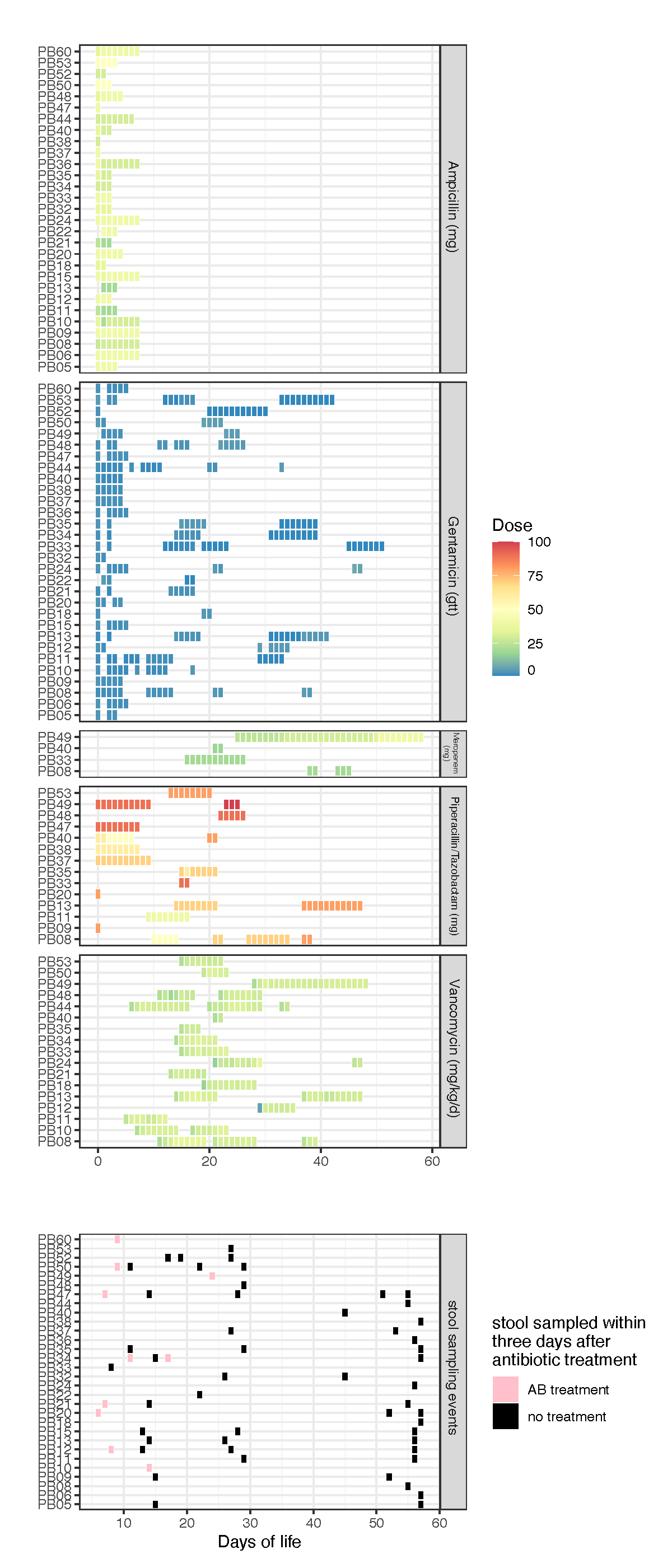

Supplement: Figure_S1.tiff [file KGMI_A_2410479_SM3708.tiff]

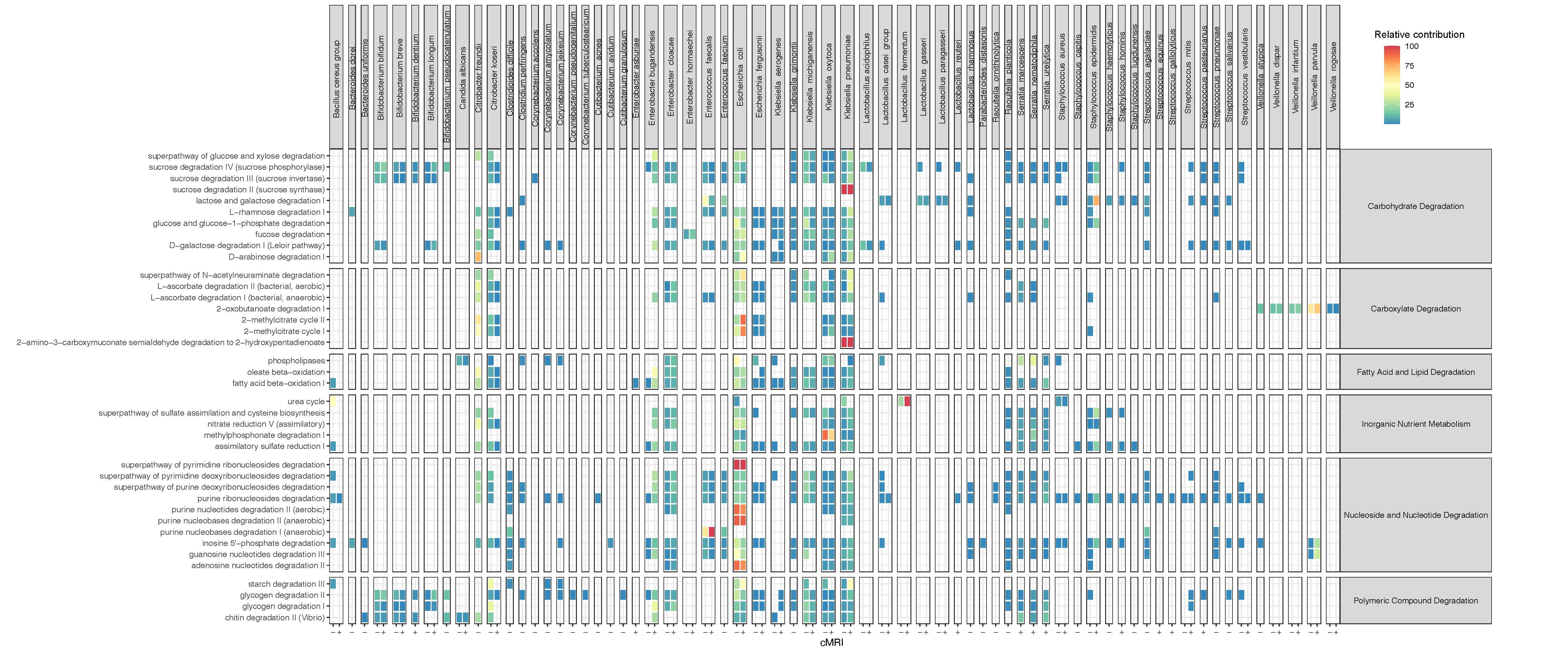

Supplement: Figure_S2.tiff [file KGMI_A_2410479_SM3707.tiff]

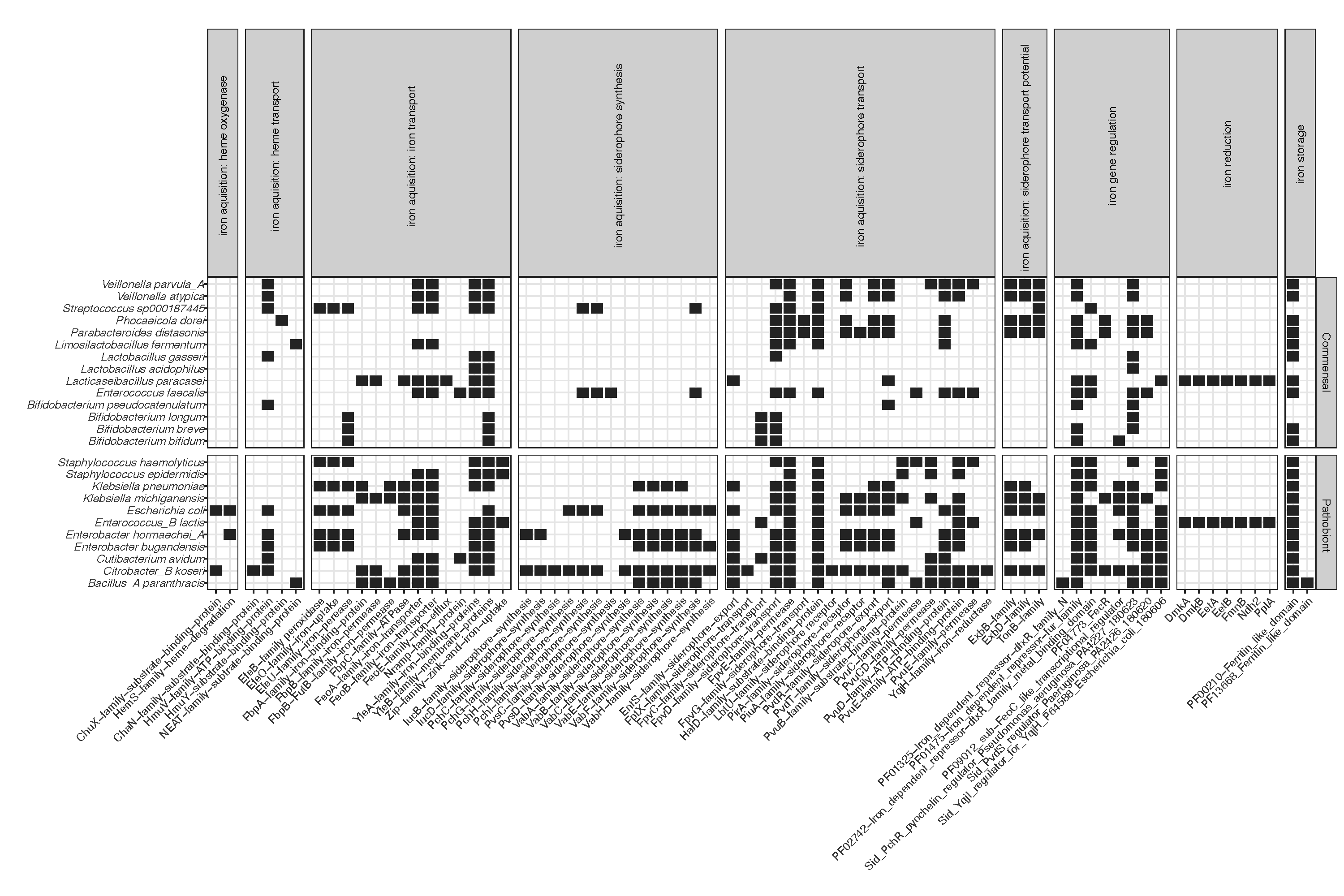

Supplement: Figure_S5.tiff [file KGMI_A_2410479_SM3706.tiff]

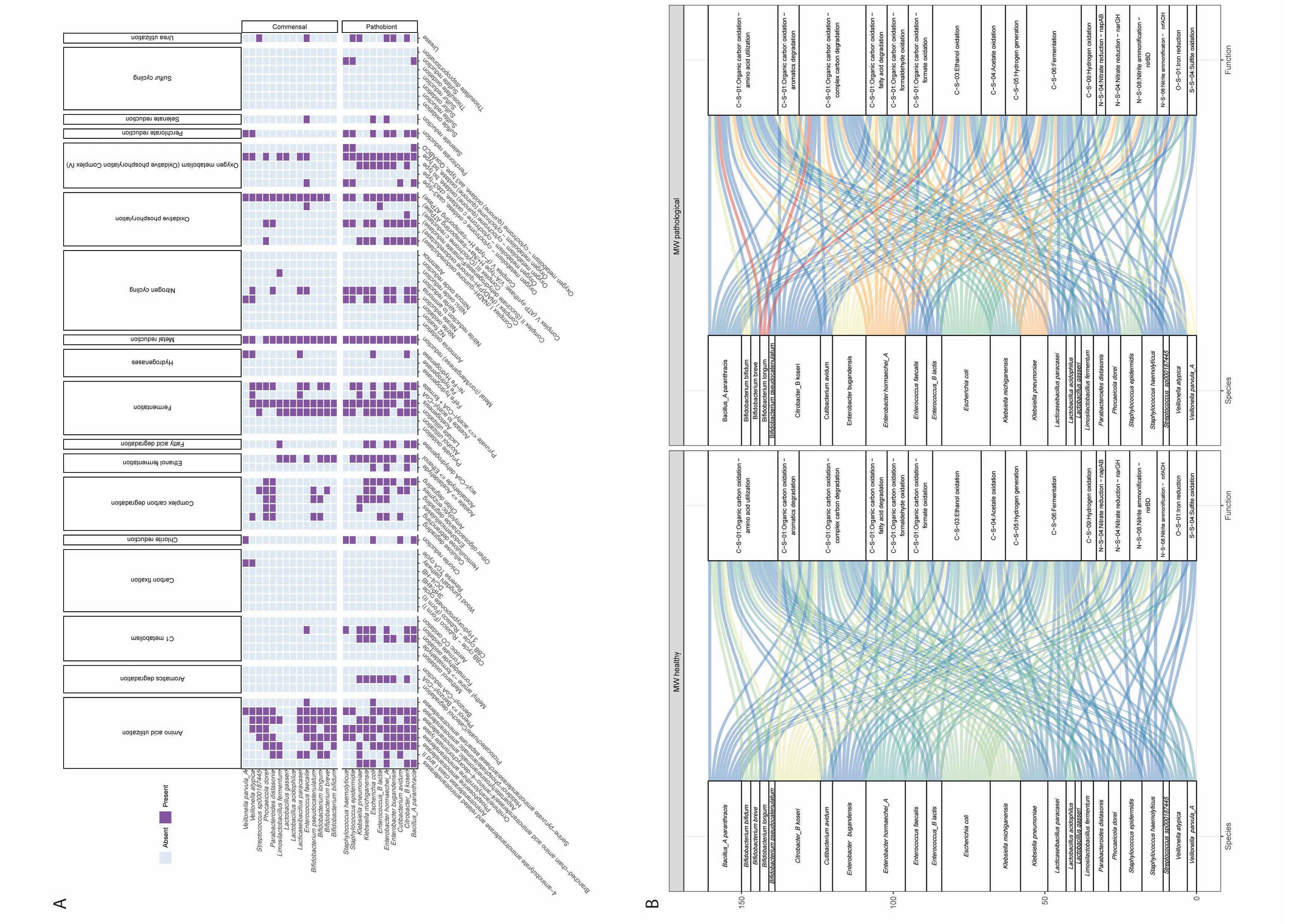

Supplement: Figure_S4.tiff [file KGMI_A_2410479_SM3704.tiff]

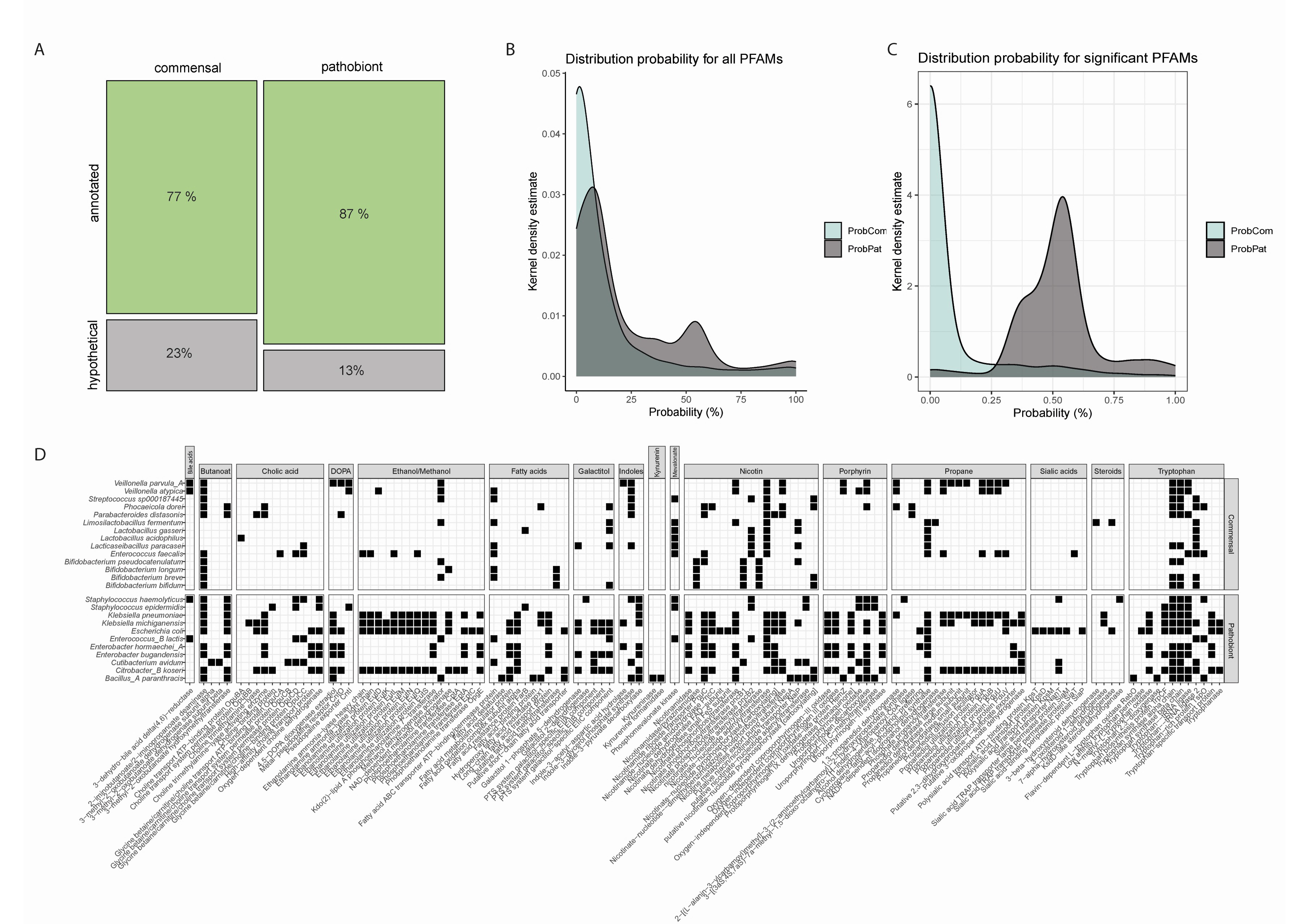

Supplement: Figure_S3.tiff [file KGMI_A_2410479_SM3703.tiff]
