# Supplementary material for: Gut microbiota genome features associated with brain injury in extremely premature infants
Source: Gut Microbes. 2024 Oct 7;16(1):2410479. doi: 10.1080/19490976.2024.2410479 (PMC11459832; doi:10.1080/19490976.2024.2410479)
Supplement: Suppl_File_1.docx [file KGMI_A_2410479_SM3705.docx]

30

25

Albumin [g/L]

20

400

200

Alkaline phosphatase [U/L]

0

40

30

20

Urea−N [mg/dl]

10

0

5

0

ART: BE [mmol/L]

−5

0 10 20 30 40

ART: pCO2 [mmHg]

70

60

50

40

7.40

7.35

7.30

ART: pH

7.25

7.20

|  |  |  |  |  |  |  |  |  |  |  |
| --- | --- | --- | --- | --- | --- | --- | --- | --- | --- | --- |
|  |  |  |  |  |  |  |  |  |  |  |
|  |  |  |  |  |  |  |  |  |  |  |
|  |  |  |  |  |  |  |  |  |  |  |
|  |  |  |  |  |  |  |  |  |  |  |
|  |  |  |  |  |  |  |  |  |  |  |
|  |  |  |  |  |  |  |  |  |  |  |
|  |  |  |  |  |  |  |  |  |  |  |
|  |  |  |  |  |  |  |  |  |  |  |

70

60

ART: pO2 [mmHg]

50

40

30

30.0

27.5

25.0

ART: SBC [mmol/L]

22.5

20.0

17.5

0.3

0.2

Bilirubin conjugated [mg/dl]

0.1

0.0

0.9

0.6

Bilirubin direkt [mg/dl]

0.3

6

4

Bilirubin total [mg/dl]

2

0

5.0

2.5

Bilirubin un−conjugated [mg/dl]

0.0

1.6

1.2

0.8

BMI

0.4

15

10

5

CAP: BE [mmol/L]

0

−5

−10

0 20

|  |  |  |  |  |  |  |  |
| --- | --- | --- | --- | --- | --- | --- | --- |
|  |  |  |  |  |  |  |  |
|  |  |  |  |  |  |  |  |
|  |  |  |  |  |  |  |  |
|  |  |  |  |  |  |  |  |
|  |  |  |  |  |  |  |  |
|  |  |  |  |  |  |  |  |
|  |  |  |  |  |  |  |  |
|  |  |  |  |  |  |  |  |
|  |  |  |  |  |  |  |  |
|  |  |  |  |  |  |  |  |
|  |  |  |  |  |  |  |  |

# Days of life

40 60

80

60

CAP: pCO2 [mmHg]

40

10

8

6

CAP: pH

4

2

30

CAP: SBC [mmol/L]

20

|  |  |  |  |  |  |  |  |
| --- | --- | --- | --- | --- | --- | --- | --- |
|  |  |  |  |  |  |  |  |
|  |  |  |  |  |  |  |  |
|  |  |  |  |  |  |  |  |
|  |  |  |  |  |  |  |  |
|  |  |  |  |  |  |  |  |
|  |  |  |  |  |  |  |  |
|  |  |  |  |  |  |  |  |
|  |  |  |  |  |  |  |  |

1.6

1.2

log(CRP [mg/dl])

0.8

0.4

1.45

1.40

1.35

Calcium ionized (BGA) [mmol/L]

1.30

1.25

1.20

0 20

# Days of life

40 60

120

110

Chloride (BGA) [mmol/L]

100

90

150

125

Glucose (BGA) [mg/dl]

100

75

4.5

4.0

Potassium (BGA) [mmol/L]

3.5

3.0

2.5

Lactate (BGA) [mmol/L]

2.0

1.5

0 20

# Days of life

40 60

145

140

Sodium (BGA) [mmol/L]

135

130

40

35

FiO2 [%]

30

25

150

100

gamma-GT [U/L]

50

50

40

30

GOT (ASAT) [U/L]

20

10

40

30

GPT (ALAT) [U/L]

20

28

26

Headsize (cm)

24

22

500

0

IL−6 [pg/ml]

0.75

0.50

Creatinin [mg/dl]

0.25

0 20

# Days of life

40 60

600

400

LDH [U/L]

200

39

36

length (cm)

33

1.2

1.0

Magnesium [mmol/L]

0.8

300

200

100

100

75

Total Volume (ml/kg)

50

25

0

Total Carbs (mg/kg/min)

100

75

Ratio parenteral (%)

50

25

0

Total Protein (g/kg/d)

160

120

Ratio enteral (%)

Total Energy (kcal/kg/d)

80

40

10

8

6

4

6

5

4

3

2

8

60

Total Fat (g/kg/d)

Parenteral Energy (kcal/kg/d)

6

40

4

20

2

0

Enteral Energy (kcal/kg/d)

6

4

2

10.0

7.5

5.0

2.5

0.0

3

2

1

0

6

4

2

0

2.0

1.5

1.0

0.5

0.0

8

6

4

2

0

150

100

Parental Fat (mg/kg/d)

50

0

0 20 40 60

Parenteral Carbs (mg/kg/min)

Parenteral Protein (g/kg/d)

0 20 40 60 0 20 40 60 0 20 40 60

Enteral Carbs (mg/kg/min)

Enteral Protein (g/kg/d)

Enteral Fat (g/kg/d)

# Days of life

cMRI age adequate and mild damage pathological and severe damage

55

Protein [g/L]

50

45

40

35

96

SaO2 [%]

|  |  |  |  |  |  |  |  |
| --- | --- | --- | --- | --- | --- | --- | --- |
|  |  |  |  |  |  |  |  |
|  |  |  |  |  |  |  |  |
|  |  |  |  |  |  |  |  |
|  |  |  |  |  |  |  |  |
|  |  |  |  |  |  |  |  |
|  |  |  |  |  |  |  |  |

94

92

200

0

−200

Triglyzeride [mg/dl]

0 20

# Days of life

40 60

10

5

0

−5

0 20

VEN: BE [mmol/L]

|  |  |  |  |  |  |  |  |
| --- | --- | --- | --- | --- | --- | --- | --- |
|  |  |  |  |  |  |  |  |
|  |  |  |  |  |  |  |  |
|  |  |  |  |  |  |  |  |
|  |  |  |  |  |  |  |  |
|  |  |  |  |  |  |  |  |
|  |  |  |  |  |  |  |  |
|  |  |  |  |  |  |  |  |
|  |  |  |  |  |  |  |  |

# Days of life

40 60

VEN: pCO2 [mmHg]

|  |  |  |  |  |  |  |  |
| --- | --- | --- | --- | --- | --- | --- | --- |
|  |  |  |  |  |  |  |  |
|  |  |  |  |  |  |  |  |
|  |  |  |  |  |  |  |  |
|  |  |  |  |  |  |  |  |
|  |  |  |  |  |  |  |  |
|  |  |  |  |  |  |  |  |
|  |  |  |  |  |  |  |  |
|  |  |  |  |  |  |  |  |

70

60

50

40

0 20 40 60

# Days of life

11

VEN: pH

10

9

8

7

6

5

VEN: pO2 [mmHg]

|  |  |  |  |  |  |  |  |
| --- | --- | --- | --- | --- | --- | --- | --- |
|  |  |  |  |  |  |  |  |
|  |  |  |  |  |  |  |  |
|  |  |  |  |  |  |  |  |
|  |  |  |  |  |  |  |  |
|  |  |  |  |  |  |  |  |
|  |  |  |  |  |  |  |  |
|  |  |  |  |  |  |  |  |
|  |  |  |  |  |  |  |  |

45

40

35

30

VEN: SBC [mmol/L]

|  |  |  |  |  |  |  |  |
| --- | --- | --- | --- | --- | --- | --- | --- |
|  |  |  |  |  |  |  |  |
|  |  |  |  |  |  |  |  |
|  |  |  |  |  |  |  |  |
|  |  |  |  |  |  |  |  |
|  |  |  |  |  |  |  |  |
|  |  |  |  |  |  |  |  |
|  |  |  |  |  |  |  |  |

30

25

20

0 20 40 60

# Days of life

1.5

1.0

0 20

weight (kg)

# Days of life

40 60
