# Supplementary material for: Gut microbiota genome features associated with brain injury in extremely premature infants
Source: Gut Microbes. 2024 Oct 7;16(1):2410479. doi: 10.1080/19490976.2024.2410479 (PMC11459832; doi:10.1080/19490976.2024.2410479)
Supplement: STORMS_Seki_2024.docx [file KGMI_A_2410479_SM3702.docx]

The STORMS checklist: Gut microbiota genome features associated with brain injury in extremely premature infants

| Number | Item: 240003582.R1 | Author summary | Item Source | Additional Guidance | Yes/No/NA | Comments or location in manuscript |
| --- | --- | --- | --- | --- | --- | --- |
| **Abstract** | | | | | | |
| 1.0 | Structured or Unstructured Abstract |  | STORMS |  | YES | Lines 25-38 |
| 1.1 | Study Design | Analytical – Observational – Longitudinal cohort | STORMS | See 3.0 for additional information on study design. |  |  |
| 1.2 | Sequencing methods | long-read Nanopore sequencing | STORMS | For example, targeted 16S by qPCR or sequencing, shotgun metagenomics, metatranscriptomics, etc. | YES |  |
| 1.3 | Specimens | extremely premature infant feces | STORMS |  | YES |  |
| **Introduction** | | | | | | |
| 2.0 | Background and Rationale | Nanopore metagenomics was used to link species-resolved taxonomy and metabolic potentials of gut microbiota to the pathology of severe brain damage in extremely premature infants | STORMS |  | YES |  |
| 2.1 | Hypotheses | Enterobacteriaceae are linked to severe brain damage during intestinal inflammation | STORMS |  | YES |  |
| **Methods** | | | | | | |
| 3.0 | Study Design | Analytical - Observational - Longitudinal cohort | STORMS | Observational (Case-Control, Cohort, Cross-sectional survey, etc.) or Experimental (Randomized controlled trial, Non-randomized controlled trial, etc.). For a brief description of common study designs see: DOI: 10.11613/BM.2014.022  If applicable, describe any blinding (e.g. single or double-blinding) used in the course of the study. | YES |  |
| 3.1 | Participants | stool from extremely premature infants (<1kg and < 28 weeks of gestation) during hospitalization. Out of 30 neonates, 6 were diagnosed with severe brain damage. | STORMS | Examples of the population of interest could be: adults with no chronic health conditions, adults with type II diabetes, newborns, etc. This is the total population to whom the study is hoped to be generalizable to. The sampling method describes how potential participants were selected from that population.  If the participants are from a substudy of a larger study, provide a brief description of that study and cite that study.  Clearly state how cases and controls are defined.  An example of relevant physiological state might be pre/post menopausal for a vaginal microbiome study; examples of stage in the life history of disease could be whether specimens were collected during active or dormant disease, or before or after treatment. | YES |  |
| 3.2 | Geographic location | Vienna, Austria | MIxS: geographic location (country and/or sea,region) | Geographic coordinates can be reported to prevent potential ambiguities if necessary. | YES |  |
| 3.3 | Relevant Dates | 2017-2024 | STORMS | Recruitment is the period in which participants are recruited for the study. In longitudinal studies, follow-up is the date range in which participants are asked to complete a specific assessment. Finally, data collection is the total period in which data is being collected from participants including during initial recruitment through all follow-ups. | YES |  |
| 3.4 | Eligibility criteria | Born before the 28th week of gestation with less than 1kg birth weight. Infants with congenital malformations, chromosomal aberrations, maternally transmitted infectious diseases, and inborn errors of metabolism were excluded. | Modified STROBE | Among potential recruited participants, how were some chosen and others not? This could include criteria such as sex, diet, age, health status, or BMI.  If there is a primary and validation sample, describe inclusion/exclusion criteria for each. | YES | Lines 96-101 |
| 3.5 | Antibiotics Usage | Antibiotics used: Ampicillin, Gentamicin, Meropenem, Piperacillin/Tazobactam, Vancomycin | STORMS | If participants were excluded due to current or recent antibiotics usage, state this here.  Other factors (e.g. proton pump inhibitors, probiotics, etc.) that may influence the microbiome should also be described as well. | YES | Figure S1 |
| 3.6 | Analytic sample size | 30 participants (24 without and 6 with severe brain damage). | STORMS | Consider use of a flow diagram (see template at https://stormsmicrobiome.org/figures). Also state sample size in abstract.  If power analysis was used to calculate sample size, describe those calculations. | YES | Lines 113-115 |
| 3.7 | Longitudinal Studies | 50 samples from infants without, and 13 samples from infants with severe brain damage; sampled between 6 and 57 days post-delivery. | STORMS | If there is loss to follow-up, discuss the likelihood that drop-out is associated with exposures, treatments, or outcomes of interest. | YES |  |
| 3.8 | Matching | / | Modified STROBE | "Matched" refers to matching between comparable study participants as cases and controls or exposed / unexposed.  Indicate whether participants were individual or frequency matched and in what ratio were they matched (e.g. 1 case to 1 control). |  |  |
| 3.9 | Ethics | approved by the ethics committee of the Medical University of Vienna (ethics number 1348/2017). Written consent was collected from parents after discussion and description of study design. | STORMS |  | YES | Lines 95-96 |
| 4.0 | Laboratory methods | University of Vienna, Division of Microbial Ecology | STORMS | Provide a reference to complete lab protocols if previously published elsewhere such as on protocols.io. Note any modifications of lab protocols and the reason for protocol modifications. | YES |  |
| 4.1 | Specimen collection | Feces was sampled with spatula+container from diapers. | MIxS: sample collection device or method; host body site | Use terms from the Uber-anatomy Ontology (https://www.ebi.ac.uk/ols/ontologies/uberon) to describe body sites in a standardized format. | YES | Lines 115-117 |
| 4.2 | Shipping | / | STORMS | Include length of time from collection to receipt by the lab and if temperature control was used during shipping. |  |  |
| 4.3 | Storage | frozen at -80° before sequencing | STORMS | State where each procedure or lot of samples was done if not all in the same place.  Include reagent/lot/catalogue #s for storage buffers. | YES | Lines 115-117 |
| 4.4 | DNA extraction | Power Soil Pro Kit & Phenol Chlorophorm extraction | MIxS: nucleic acid extraction | If any DNA quantification methods were used prior to DNA amplification or at the pooling step of library preparation, state so here. | YES | Lines 119-132 |
| 4.5 | Human DNA sequence depletion or microbial DNA enrichment | / | STORMS |  |  |  |
| 4.6 | Primer selection | / | MIxS: pcr primers |  |  |  |
| 4.7 | Positive Controls | / | STORMS | If used, should be deposited under guidance provided in the 8.X items. | YES | Lines 119-132 |
| 4.8 | Negative Controls | Kit & Buffer / Water controls for sequencing | STORMS | If used, should be deposited under guidance provided in the 8.X items. | YES | Lines 119-132 |
| 4.9 | Contaminant mitigation and identification | Kit & Buffer controls; MAGs with > 5% contamination were excluded | STORMS | Includes filtering of reagents and other steps to minimize contamination. It is relevant to state whether the specimens of interest have low microbial load, which makes contamination especially relevant. | YES | Lines 246-250 |
| 4.10 | Replication | / | STORMS | Replication may be biological (redundant biological specimens) or technical (aliquots taken at different stages of analysis) and used in extraction, sequencing, preprocessing, and/or data analysis. |  |  |
| 4.11 | Sequencing strategy | Short-read meteganomics (Illumina) and long-read nanopore sequencing | MIxS: sequencing method | For amplicon sequencing (for example, 16S variable region), state the region selected. State the model of sequencer used. |  | Lines 113-159 |
| 4.12 | Sequencing methods | Promethion P24 (Oxford Nanopore) and NovaSeq 6000 | STORMS | These include read length, sequencing depth per sample (average and minimum), whether reads are paired, and other parameters. |  | Lines 118-132 |
| 4.13 | Batch effects | / | STORMS | Sources of batch effects include sample collection, storage, library preparation, and sequencing and are commonly unavoidable in all but the smallest of studies. |  |  |
| 4.14 | Metatranscriptomics | / | STORMS | Provide details on any internal standards which may have been used as well as parameters and versions of any software or databases used. |  |  |
| 4.15 | Metaproteomics | / | STORMS | Provide details on any internal standards which may have been used as well as parameters and versions of any software or databases used. |  |  |
| 4.16 | Metabolomics | / | STORMS | Provide details on any internal standards which may have been used as well as parameters and versions of any software or databases used. |  |  |
| 5.0 | Data sources/  measurement | clinical parameters of neonates were continuously monitored | MIxS: host disease status | State any sources of potential bias in measurements, for example multiple interviewers or measurement instruments, and whether these potential biases were assessed or accounted for in study design.  Use terms from a standardized ontology such as the Experimental Factor Ontology (https://www.ebi.ac.uk/efo/) to describe variables of interest in a standardized format. | YES | Table 2 |
| 6.0 | Research design for causal inference | / | STORMS | For causal inference, this item refers to describing the assumptions that would be required to draw causal inferences from observational data. See Vujkovic-Cvijin, I., Sklar, J., Jiang, L. et al. Host variables confound gut microbiota studies of human disease. Nature 587, 448–454 (2020). https://doi.org/10.1038/s41586-020-2881-9 for more details on confounding in observational microbiome studies.  For example, hypothesized confounders may be controlled for by multivariable adjustment. Consider using a directed acyclic graph (DAG) to describe your causal model and justify any variables controlled for. DAGs can be made using [www.dagitty.net](http://www.dagitty.net/). |  |  |
| 6.1 | Selection bias | / | STORMS | Selection bias can occur when some members of the target study population are more likely to be included in the study/final analytic sample than others. Some examples include survival bias (where part of the target study population is more likely to die before they can be studied), convenience sampling (where members of the target study population are not selected at random), and loss to follow-up (when probability of dropping out is related to one of the things being studied). |  |  |
| 7.0 | Bioinformatic and Statistical Methods | abundance corrected reads are depicted as percentages | STORMS | If a variable is analyzed using different transformations, state rationale for the transformation and for each analyses which version of the variable is used.  In case of any complex or multistep transformations, give enumerated instructions for reproducing those transformations. | YES | Lines 140-141 |
| 7.1 | Quality Control | sufficient DNA concentration was assessed using the HSdsDNA Assay Kit (Thermo Fisher) on a Qubit 4 Fluorometer | MIxS: sequence quality check | If samples were excluded based on quality or read depth, list the criteria used, the number of samples excluded, and the final sample size after quality control. | YES | Lines 123-124 |
| 7.2 | Sequence analysis | read based processing of sequencing data and de-novo assembly of MAGs is described in detail in the manuscript | MIxS: feature prediction; similarity search method |  | YES | Lines 118-159 |
| 7.3 | Statistical methods | Statistical analysis (Student’s T-test, ANOVA, repeated measures ANOVA, Wilcoxon Test, and Fisher’s exact test) were performed in R version 4.0 and the R package rstatix version 0.7.0. Hierarchical clustering was performed with ComplexHeatmap on log-transformed abundance counts, and DESeq2 was used for differential abundance testing of microbial species. All p-values were adjusted using Bonferroni’s method. Data was visualized via R version 4.0 and R package ggplot2 version 3.3.3 | Modified STROBE | Describe any statistical tests used, exploratory data analysis performed, dimension reduction methods/unsupervised analysis, alpha/beta metrics, and/or methods for adjusting for measurement bias.  If multiple statistical methods are possible, discuss why the methods used were selected.  If a multiple hypothesis testing correction method was used, describe the type of correction used.  State which taxonomic levels are analyzed. | YES | Lines 160-166 |
| 7.4 | Longitudinal analysis | reads were averaged per participant | STORMS |  | YES | Figure S1 |
| 7.5 | Subgroup analysis | / | STROBE |  |  |  |
| 7.6 | Missing data | / | STROBE | "Missing data" refers to participant measurements such as covariates, exposures, outcomes, or time points that should have been collected but were not, not to zeros in taxonomic abundance tables or data points not applicable to that observation. |  |  |
| 7.7 | Sensitivity analyses | / | STROBE |  |  |  |
| 7.8 | Findings | All p-values were adjusted using Bonferroni’s method. Findings were significant if corrected p-value < 0.05 | STORMS | For example, false discovery rate with total number of tests, effect size threshold, significance threshold, microbes of interest. | YES | Lines 165-166 |
| 7.9 | Software | Guppy (Reads basecalling); HUMAnN3 (short-read metagenomic data processing) including bowtie2 for mapping with DIAMOND; assembly of nanopore reads with flye, pollishing of assembly with racon/medaka & minimap2 (aligner), cutadapt (trimming), metabat (binning), QUAST and CheckM (Quality control), GTDBtk (taxonomic classification) Prokka (functional annotation), dRep (derepliocation), ABRicate (screening for antimicrobial resistances), FeGenie (screen for iron related genes), METABOLIC (classification of metabolic capabilites of dereplicated genomes) | Modified STREGA | Installed packages, add-ons or libraries should be stated and cited in addition to the software used.  All parameters employed that differ from the default of that software/version should be provided.  This is in addition to, not a replacement for, publishing of code as outlined in the section Reproducible Research. | YES |  |
| 8.0 | Reproducible research | Further information and requests for resources and reagents should be directed to and will be fulfilled by the lead contacts David Berry ([david.berry@univie.ac.at](mailto:david.berry@univie.ac.at)) and David Seki (david.seki@univie.ac.at); Deposition of materials under BioProject ID: PRJNA1112760 | STORMS | Any protected information that has been excluded or provided under controlled access should be listed along with any relevant data access procedures. "On request from authors" is not sufficiently detailed; formal data access procedures and conditions should be defined.  If data are unavailable, state so clearly.  Consider using a specialized rubric for reproducible research (such as:<https://mbio.asm.org/content/9/3/e00525-18.short)>.  Consider preregistering the study protocol (such as o[n osf.](http://osf.io/)io or<https://plos.org/open-science/preregistration/).> | YES |  |
| 8.1 | Raw data access | Deposition of materials under BioProject ID: PRJNA1112760 | STORMS | Robust, long-term databases such as those hosted by NCBI and EBI are preferred. If using a private repository, provide rationale. | YES |  |
| 8.2 | Processed data access | Deposition of materials under BioProject ID: PRJNA1112760 | STORMS | Unfiltered data should be provided.  Robust, long-term databases such as those hosted by NCBI and EBI-EMBL are preferred. Repositories like zenodo (https://zenodo.org/) or publisso (https://www.publisso.de/en/working-for-you/doi-service/)  can be used to provide a DOI and long-term storage for processed datasets, even those which cannot be published openly. | YES |  |
| 8.3 | Participant data access | Requests for metadata should be directed to the lead contacts David Berry ([david.berry@univie.ac.at](mailto:david.berry@univie.ac.at)) and David Seki (david.seki@univie.ac.at) , who can provide data linked to anonymized Patient identifiers upon request | STORMS | If re-categorized, transformed, or otherwise derived variables were used in the analysis, these variables or code for deriving them should be provided.  Examples of how participant data can be matched to microbiome data are: using the same set of anonymized identifiers, or using different anonymized identifiers but providing a map.  Provided data should be sufficient to independently replicate the current analysis. | YES |  |
| 8.4 | Source code access | Further information and requests for code should be directed to and will be fulfilled by the lead contact David Seki (david.seki@univie.ac.at). | STORMS | If a standard or formalized workflow was employed, reference it here. | YES |  |
| 8.5 | Full results | each result is provided in the flow of the text and within figures of the manuscript | STORMS | For example, any fold-changes, p-values, or FDR values calculated, provided as a spreadsheet.  Use a machine-readable, plain-text format such as csv or tsv. | YES | Table 1; Table 2 |
| **Results** | | | | | | |
| 9.0 | Descriptive data | fecal microbiota of extremely premature infant with and without severe brain damage | STROBE | Typically reported in a table included in the paper or as a supplementary table. Indicate number of participants with missing data for each variable of interest.  This includes environmental and lifestyle factors that may affect the relationship between the microbiome and the condition of interest. Participant diet and medication use should be summarized, if known.  At minimum, age and sex of all participants should be summarized. | YES |  |
| 10.0 | Microbiome data | Hierarchical clustering of short-read samples did not indicate grouping due to neurophysiological outcome. Also, PERMANOVA as well as PCA revealed no significant relationship between cMRI diagnosis or days of life post-delivery on microbiome composition (PERMANOVA, p.adj = 0.49). Likewise, no significant differences in observed alpha diversity were detected between infants with and without severe brain damage (t test, p.adj = 0.113). However, MAGs assembled from long-read data show, that putative pathobionts were elevated in infants with severe brain injury, and that pathobiont MAGs were enriched in several protein families, including L-lactate permease and both subunits for nitrite reductase, which may be important to survival in the GIT under chronic inflammatory conditions. | STORMS | This includes measures of diversity as well as relative abundances. These descriptive findings should be reported both for the sample overall and for individual groups. | YES |  |
| 10.1 | Taxonomy | Community composition of shor-read metagenomic data was estimated with MetaphlAn4. MAGs assembled from long-read nanopore reads were taxonomically classified using GTDBtk. | STORMS | If not using full taxonomic hierarchy, make sure it is clear whether names stated are species, genera, family, etc.  Italicize genus/species pairs. Consult journal guidelines or standardized references on taxonomic nomenclature. For instance,<https://wwwnc.cdc.gov/eid/page/scientific-nomenclature> | YES | Table 1 |
| 10.2 | Differential abundance | DESeq2 was used for differential abundance testing of microbial species. Among prevalent species, Enterobacter bugandensis had the largest average enrichment in infants with severe brain damage and Enterococcus faecalis the largest average depletion, although these trends were not significant after correction for multiple testing. | STORMS | If there are more than two groups, include omnibus (multigroup) test results if applicable to the research question.  If applicable, reported effect sizes should include a measure of uncertainty such as the confidence interval. | YES | Lines 163-165 |
| 10.3 | Other data types | The number of functional pathways in short-reads was assessed via HUMAnN3. We find that altered functional potential and reduced functional redundancy are associated with brain damage. Furthermore, we assembled genomes from metagenomic reads, yielding 25 unique, highly complete (> 90%), and high-quality (< 5% contamination) bacterial MAGs after dereplication. In these genomes, we identify key genomic features of pathobionts and commensals. Pathobiont traints include elevated potential for nitrate reduction and iron scavenging. | STORMS |  | YES |  |
| 10.4 | Other statistical analysis | / | STORMS | This could include subgroup analysis, sensitivity analyses, and cluster analysis.  Visualizations should be easily interpretable and colorblind-friendly. The caption and/or main text should provide a detailed description of visualizations for visually-impaired readers. |  |  |
| **Discussion** | | | | | | |
| 11.0 | Key results | Summarise key results with reference to study objectives | STROBE |  |  |  |
| 12.0 | Interpretation | we adressed gut microbiome composition and metabolic traits that are associated with severe brain damage in extremely premature infants. By using and cross validating several metagenomic approaches, we find that pathobionts are cumulatively more abundant in premature infants with neuropathological outcomes. They possess a versatile genomic potential that facilitates survival during chronic inflammation, including multiple respiratory pathways, many antibiotic resistance genes, and several means of iron acquisition. | STROBE | Define or clarify any subjective terms such as "dominant," "dysbiosis," and similar words used in interpretation of results.  When interpreting the findings, consider how the interpretation of the findings may be summarized or quoted for the general public such as in press releases or news articles.  If causal language is used in the interpretation (such as "alters," "affects," "results in," "causes," or "impacts"), assumptions made for causal inference should be explicitly stated as part of 6.0 and 13.0.  Distinguish between function potential (ie inferred from metagenomics) and observed activity (ie metatranscriptomic, metabolomic, proteomic) if discussing microbial function. | YES |  |
| 13.0 | Limitations | since long-read nanopore sequencing is a failry unestablished method, we used short-read metagenomics additionally to polish assembly of our MAGs | STROBE | Also consider limitations resulting from the methods (especially novel methods), the study design, and the sample size. | YES |  |
| 13.1 | Bias | early-life assembly of infant gut microbiota is a highly dynamic process, and even though we have obtained a comparatively large dataset, full understanding of this process will require daily sampling. | STORMS | May include sampling method, representativeness of study participants, or potential confounding. | YES |  |
| 13.2 | Generalizability | We expect our results and conclusions to generalize to extremely premature infants in hospitalized settings, and they may be compared to healthy term-born infants. | STROBE | To what populations or other settings do you expect the conclusions to generalize? | YES |  |
| 14.0 | Ongoing/future work | the molecular details of how specific Enterobacteriaceae such as Klebsiella pneumoniae may become dominant in the infant gut. | STORMS |  | YES |  |
| **Other information** | | | | | | |
| 15.0 | Funding | This project was funded by an inter-university cluster project grant between the University of Vienna and the Medical University of Vienna (“PreMiBraIn”), the European Research Council (Starting grant: FunKeyGut 741623), and the Austrian Science Fund (FWF; P27831-B28, FG 29), led by David Berry. Lindsay J. Hall is supported by: Wellcome Trust Investigator Awards no. 220876/Z/20/Z. | STROBE |  | YES | Lines 601-605 |
| 15.1 | Acknowledgements | We would like to thank Gudrun Kohl, Jasmin Schwarz and Hester Schodterer for technical assistance with sample processing in the JMF laboratories. | STORMS | For general guidelines on authorship, see [http://www.icmje.org](http://www.icmje.org/) and<https://www.elsevier.com/authors/journal-authors/policies-and-ethics/credit-author-statement> | YES | Lines 596-605 |
| 15.2 | Conflicts of Interest | we declare no competing interest | STORMS |  | YES | Lines 611-612 |
| 16.0 | Supplements | Figure S1 – S5 | STORMS |  | YES |  |
| 17.0 | Supplementary data | Figure S1-S5 | STORMS | Depending on the analysis performed, examples of the supplemental results included could be mean relative abundance, differential abundance, raw p-value, multiple hypothesis testing-adjusted p-values, and standard error.  All discussed taxa should include the taxonomic level (e.g. class, order, genus). | YES |  |
